# Metabolic engineering of *Yarrowia lipolytica* targeting bottlenecks to boost D-Pantothenic acid biosynthesis

**DOI:** 10.1186/s40643-026-01009-4

**Published:** 2026-01-27

**Authors:** Xing-Kai Li, Nuo Zhang, Hai-Peng Li, Zheng-Yu Huang, Gao-Yue Niu, Chen-Yi Sun, Jian-He Xu

**Affiliations:** 1 State Key Laboratory of Bioreactor Engineering, Shanghai Collaborative Innovation Centre for Biomanufacturing, College of Biotechnology, East China University of Science and Technology, Shanghai, 200237 China; 2 Shanghai Bioforany Biotechnology Corporation Limited, 471 Guiping Road, Shanghai, 200233 China

**Keywords:** D-Pantothenic acid, Vitamin B_5_, *Yarrowia lipolytica*, Metabolic engineering, Multi-cofactor engineering, Fed-batch fermentation

## Abstract

D-Pantothenic acid (DPA), also known as vitamin B_5_, is a water-soluble organic acid, widely applied in foods, feeds, cosmetics, and medicines. Although numerous and rapidly developing cell factories have been established for DPA biosynthesis, there has been no report of any attempts to engineer *Yarrowia lipolytica* to synthesize DPA. To explore further possibilities in DPA biosynthesis, we tried to employ systematic metabolic engineering strategies to identify and break the potential bottlenecks in DPA biosynthesis by* Y. lipolytica*. By improving the rate-limiting steps of the DPA biosynthesis pathway, weakening the strongly competitive pathways, and enhancing the multiple cofactor supplies, a robust *Y. lipolytica* cell factory for DPA biosynthesis was successfully constructed. Consequently, the resulting strain DPA34 produced 2.18 g/L DPA in a 5-L bioreactor, representing the first report of DPA production to date in *Y. lipolytica.* This work is believed to facilitate the development of *Y. lipolytica* for sustainable manufacturing of vitamin B_5_ and its derivatives.

**Graphical abstract:**

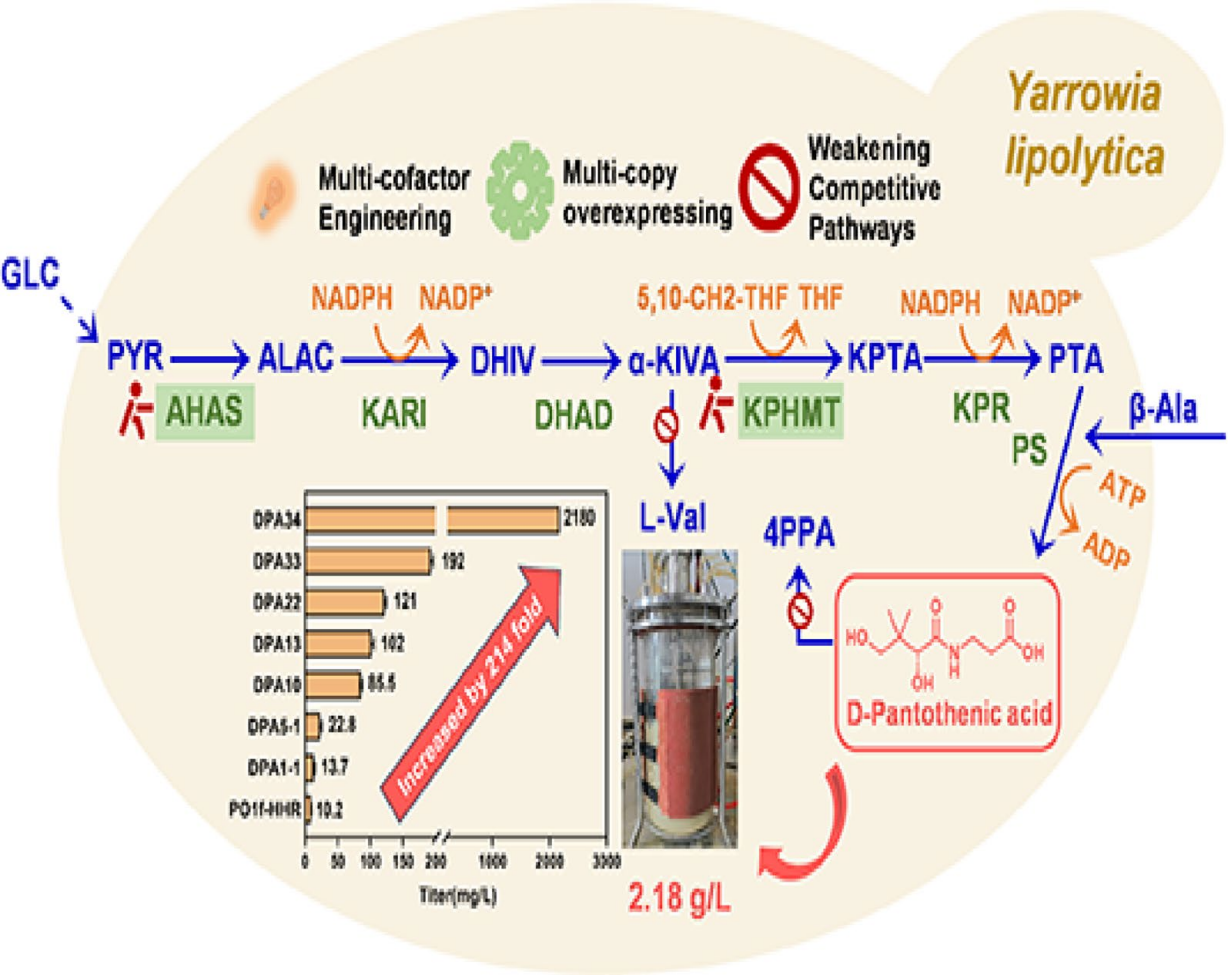

**Supplementary Information:**

The online version contains supplementary material available at 10.1186/s40643-026-01009-4.

## Introduction

D-Pantothenic acid (DPA), also known as vitamin B_5_, is a water-soluble organic acid, which plays an important role in the biosynthesis of coenzyme A (CoA) and the acyl carrier protein (ACP), as well as in the metabolism of essential nutrients including proteins, fats, and carbohydrates (Barrit et al. 2024; Eggersdorfer et al. 2012; Miallot et al. 2023). DPA has also found wide applications in various industries, including foods (Revuelta et al. 2016), feeds (Zehra and Khan 2018), cosmetics (Pastor-Nieto et al. 2021), and medicines (Xu et al. 2020). Currently, DPA production primarily depends on chemical synthesis. However, it is environmentally unfriendly because of large quantities of toxic gases and wastewater involved in the production process (Rowicki et al. 2006). Microbial fermentation shows a great potential in the sustainable production of natural products, since it can utilize renewable bioresources (Francois et al. 2020; Zhao et al. 2023; Acevedo-Rocha et al. 2019). Hence, the construction of a robust cell factory for DPA biosynthesis becomes a hot topic.

In recent years, many researchers have established DPA cell factories using various microorganisms. Among these cell factories, some of researchers chose prokaryotic hosts such as *Escherichia coli* (Zhang et al. 2019; Zhao et al. 2025a), *Bacillus subtilis* (Yuan et al. 2023; Mao et al. 2024), *Bacillus megaterium* (Tadi et al. 2022), and *Corynebacterium glutamicum* (Hüser et al. 2005; Su et al. 2023). In particular, *E. coli* has demonstrated a strong potential for DPA production, achieving the highest DPA titer of 148 g/L with β-Ala supplementation and 65.1 g/L without β-Ala supplementation (Zhang et al. 2025a). A series of metabolic engineering strategies have been adopted to enhance DPA production in *E. coli*, such as competitive pathway inhibition (Zhang et al. 2019), key enzyme modification (Cai et al. 2023; Zhang et al. 2025a; Qiu et al. 2025), transcriptomic analysis (Song et al. 2024), cofactor engineering (Zou et al. 2022; Qiu et al. 2025), and fermentation optimization (Zou et al. 2021).

However, advances in synthetic biology have facilitated the production of diverse natural products in eukaryotic hosts, particularly in yeasts (Zhang et al. 2025b; Xu et al. 2023). To date, biosynthesis of DPA has been achieved in *Saccharomyces cerevisiae* (Guo et al. 2023), and a DPA-producing strain of *S. cerevisiae* was successfully constructed by screening seven key genes derived from various species and optimizing the copy number of pathway modules, ultimately reaching a titer of 4.1 g/L in a 1-L bioreactor.

**Fig. 1:**
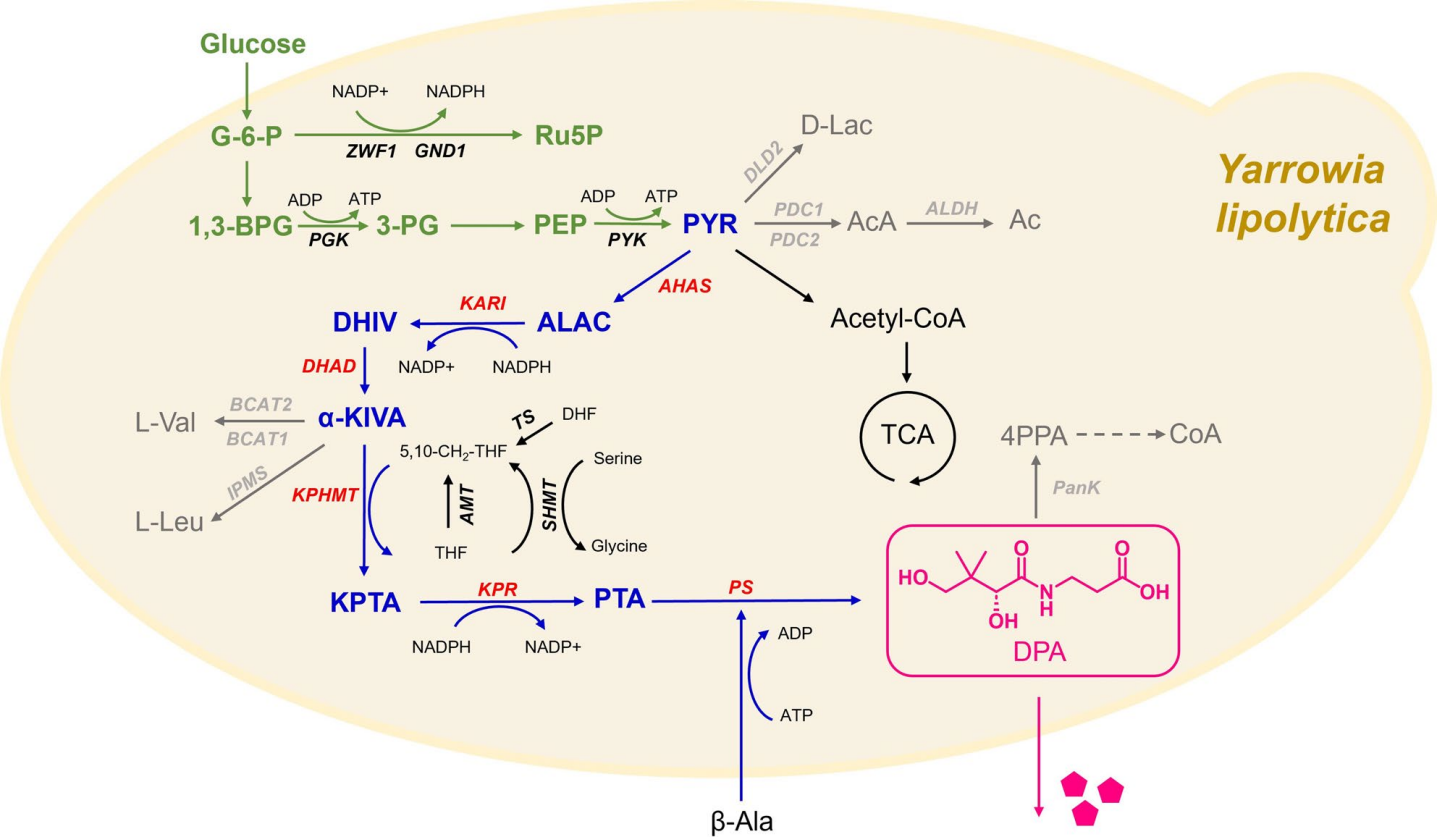
Biosynthetic pathways for DPA production in *Y. lipolytica*. The target product (DPA, pink), main biosynthesis pathway (blue arrows), key enzymes for D-pantothenic acid (red), central metabolism (green arrows), and competing pathways (grey arrows) are indicated. AHAS, acetohydroxyacid synthase; KARI, ketol-acid reductoisomerase; DHAD, dihydroxyacid dehydratase; KPHMT, ketopantoate hydroxymethyltransferase; KPR, ketopantoate reductase; PS, pantothenate synthetase; ZWF1, glucose-6-phosphate dehydrogenas; GND1, 6-phosphogluconate dehydrogenase; TS, thymidylate synthase; SHMT, hydroxymethyltransferase; AMT, aminomethyltransferase; PGK, phosphoglycerate kinase; PYK, pyruvate kinase; BACT1/BACT2, branched-chain amino acid aminotransferase; IMPS, 2-isopropylmalate synthase; DLD2, D-2-hydroxyglutarate–pyruvate transhydrogenase; PDC1/PDC2, pyruvate decarboxylase; ALDH, aldehyde dehydrogenase; PanK, pantothenate kinase; PYR, pyruvate; ALAC, 2-Acetolactate; DHIV, 2,3-Dihydroxy-isovalerate; α-KIVA, α-Ketoisovalerate; KPTA, ketopantoic acid; PTA, pantoic acid; 4PPA, 4-phosphate pantothenic acid

*Yarrowia lipolytica*, an unconventional yeast, has produced a range of organic acids such as succinic acid (Zhong et al. 2024), L-malic acid (Wang et al. 2024), itaconic acid (Fu et al. 2024), and chlorogenic acid (He et al. 2025), demonstrating an enormous potential for producing organic acids. However, there has been no report of attempts to engineer *Y. lipolytica* to synthesize DPA from simple carbon sources. Therefore, we investigated the potential bottlenecks for DPA synthesis in *Y. lipolytica* to progressively enhance the DPA production (Fig. 1).

Herein, we initiated our research from an engineered *Y. lipolytica* strain, Po1f-HHR (Xu et al. 2024 ), with a significantly improved homologous recombination efficiency, aiming at targeting and breaking its bottlenecks to boost DPA biosynthesis through systematic metabolic engineering approaches. First, identification of rate-limiting steps and combination of key enzymes together achieved synergistic optimization to enhance the DPA titer. Then, weakening of the strongly competitive pathways redirected the metabolic flux for further enhancement of DPA synthesis. By enhancing the multiple cofactor supplies, we improved the efficiency of entire DPA synthesis pathway, achieving a DPA titer of 192 mg/L in shake-flask cultures. Ultimately, the best strain DPA34, achieved a DPA titer of 2.18 g/L through fed-batch fermentation in a 5-L bioreactor, representing the first report of DPA production in the *Y. lipolytica* cell factory.

## Materials and methods

### Strains and plasmids

*Escherichia coli* DH5α was applied for cloning and plasmid construction. Strain Polf-HHR, whose homologous recombination efficiency was improved by disrupting the *KU70* gene and co-overexpressing *ScRAD52* and *ScRAD59* genes (Xu et al. 2024), was chosen as the initial strain for metabolic engineering in this work. The strains and plasmids used in this study are summarized in Tables S1 and S2.

### Culture media and conditions

*E. coli* DH5α was cultured in Luria − Bertani broth (tryptone 10 g/L, yeast extract 5 g/L, and NaCl 10 g/L) supplemented with 100 mg/L ampicillin at 37 °C under constant shaking at 200 rpm. All the *Y. lipolytica* strains were cultivated at 30 °C with shaking at 220 rpm. YPD medium containing 10 g/L yeast extract, 20 g/L peptone and 20 g/L glucose, and synthetic complete (SC) medium containing 0.67% yeast nitrogen base with (NH_4_)_2_SO_4_, appropriate amino acids, 2% glucose and 2.5 g/L β-alanine, were used for the cultivation of *Y. lipolytica*. YNB medium (6.7 g/L yeast nitrogen base plus 20 g/L glucose) was used for the selection of recombinant strains. YPD with 1 g/L of 5-fluoroorotic acid (5-FOA) used to remove the URA3 genetic maker after integration for the next round of transformation.

### DNA manipulation

All the primers used in this study are listed in Table S3. All the genes of *Y. lipolytica* origin were cloned directly from its genomic DNA using PrimerSTAR Max DNA polymerase (Takara, Dalian, China). Other heterologous genes were synthesized by Genscript (Nanjing, China) after codon optimization. To ensure stable gene expression, all expression cassettes were integrated into the chromosome by CRISPR-Cas9 genome editing (Xu et al. 2024).

### Transformation of *Y. lipolytica*

Gene expression cassettes were integrated into the *Y. lipolytica* genome using the Frozen-EZ Yeast Transformation II Kit (Zymo Research, Orange, CA, USA). The genomic DNA of positive clones was extracted using MightyPrep reagent for DNA (Takara, Dalian, China) and verified through PCR.

### Shake-flask and fed-batch fermentation

The engineered strains were cultivated in 5 mL YPD medium at 30 °C and 220 rpm for approximately 16 h. For shake-flask fermentation, cultures were inoculated into 250 − mL flasks containing 50 mL of SC medium supplemented with 2.5 g/L β-alanine, at an initial OD₆₀₀ of 0.2, and incubated at 30 °C and 220 rpm for 144 h.

For fed-batch fermentation, 1% (v/v) of the seed culture was transferred to 200 mL YPD medium and incubated under the same conditions for approximately 16 h. Subsequently, 10% of this culture was inoculated into BSM medium in a 5-L bioreactor to initiate the fed-batch fermentation. Basal salt medium (BSM): 40 g/L glucose, 1.14 g/L CaSO_4_⋅2H_2_O, 12.2 g/L MgSO_4_⋅7H_2_O, 14.3 g/L K_2_SO_4_, 3.3 g/L KOH, 21 mL/L H_3_PO_4_, 10 g/L (NH_4_)_2_SO_4_, 2.5 g/L β-alanine, and 12 mL/L PTM1 (Yuanye, Shanghai, China).

The fermentation process consisted of two phases. The growth phase (Phase I) was initiated in BSM medium to promote initial cell growth and lasted for approximately 16–20 h. After glucose was depleted, 800 g/L glucose containing 1.2% (*v*/*v*) PTM1 and 40 g/L β-alanine were gradually fed in the production phase (Phase II). The pH was maintained at 6.0 by adding 30% (*v*/*v*) ammonia solution, and the temperature was controlled at 30 °C. The dissolved oxygen (DO) concentration was maintained at approximately 30% saturation by adjusting the stirring speed (200–1000 rpm) while the airflow rate was kept constant (3.0 L/L/min).

### Analytical methods

Dry cell weight of *Y. lipolytica* was measured by weighing the cells from 1 mL of fermentation broth after dried at 85 °C for 48 h. After fermentation, the fermentation broth (1 mL) was centrifuged at 9,000 × *g* for 10 min. For D-Pantothenic acid (DPA) analysis, the supernatant of the fermentation broth was collected, filtered through a 0.22-μm pore-size membrane and subsequently loaded onto HPLC (Shimadzu-LC2030, Kyoto, Japan) with a Hypersil ODS-2 C18 column (250 × 4.6 mm, 5 μm) to analyze the DPA concentration. The chromatographic conditions were as follows: column temperature, 35 °C; mobile phase, 20 mM potassium dihydrogen phosphate and acetonitrile (95:5, v/v); flow rate, 1.0 mL/min. DPA was detected at 200 nm using a ultraviolet (UV) detector. The amino acid content was determined after the precolumn derivatization of 2,4-dinitrofluorobenzene (DNFB) . The pyruvate content was measured by HPLC using an Aminex HPX-87H column (300 mm × 7.8 mm, Bio-Rad, Hercules, CA, USA), with a UV detector at 210 nm. The column temperature was set at 35 °C and 10 mM H_2_SO_4_ was used as the mobile phase at a flow rate of 0.5 mL/min. Cellular NADPH/NADP^+^ were quantified using the CheKine NADP^+^/NADPH assay kit (catalog no. WST-8; Abbkine). Samples were individually extracted with the NADPH or NADP^+^ extraction buffers and all subsequent steps were performed according to the manufacturer’s instructions. All assays were performed at least in triplicate.

## Results and discussion

### Screening pantothenate synthetase to enhance DPA synthesis

**Fig. 2:**
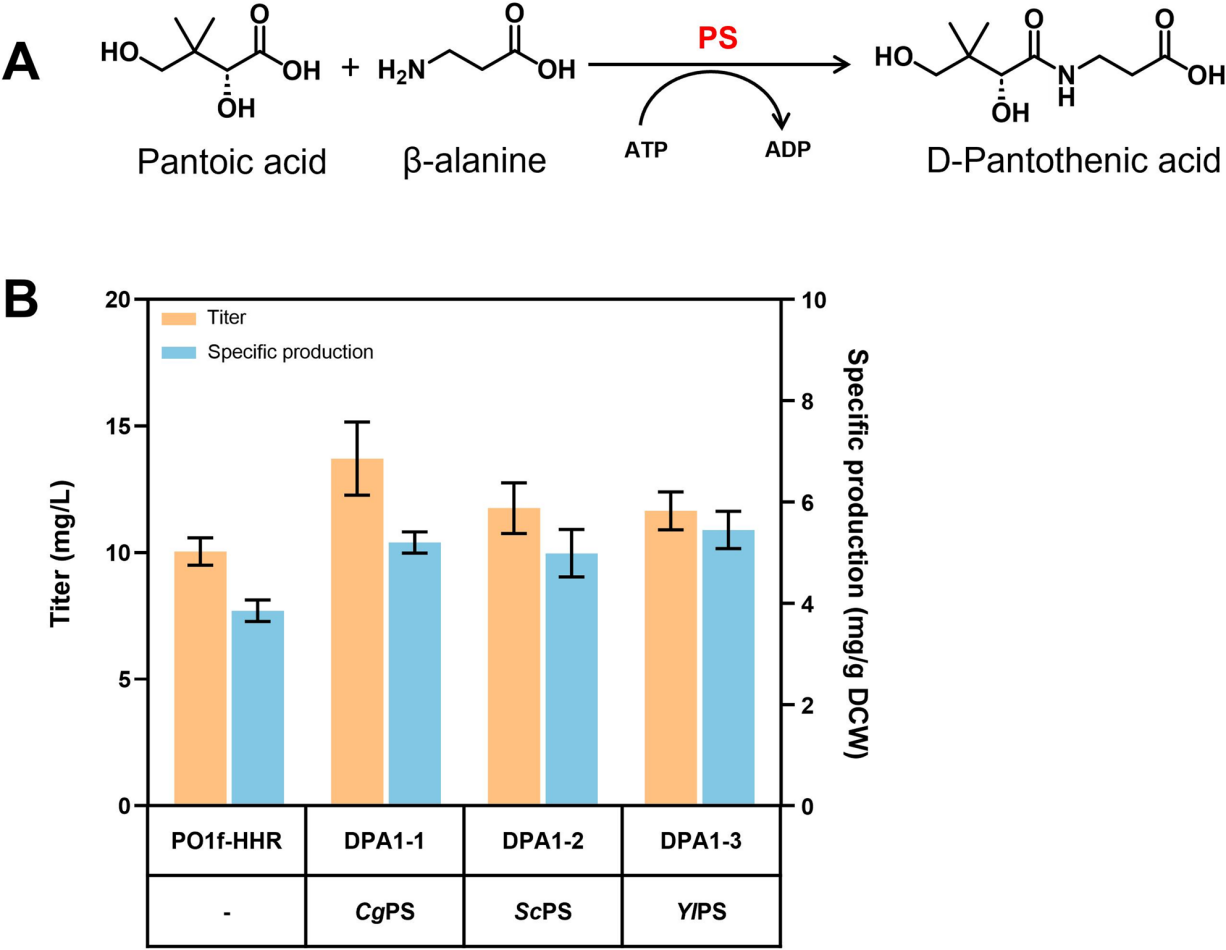
Screening of Pantothenate Synthetases (PS) enhances DPA synthesis in *Y. lipolytica*. (**A**) The condensation reaction catalyzed by PS. (**B**) DPA titer and specific production of the strains with PSs from three sources. *Yl*PS from *Y. lipolytic*; *Sc*PS from *S. cerevisiae*; *Cg*PS from *C. glutamicum*. The data represent the means ± standard deviations (*n* = 3)

The initial strain PO1f-HHR produced only 10.2 mg/L DPA (Fig. 2B), which is extremely low. Pantothenate synthetase (PS) catalyzes the condensation of β-alanine with pantoic acid (PTA) to synthesize DPA (Fig. 2A), and PS is widely recognized as a key rate-limiting enzyme in DPA biosynthesis pathway (Zhang et al. 2019; Cai et al. 2023; Guo et al. 2023; Qiu et al. 2025). Hence, PS may be a key bottleneck and an efficient PS will facilitate the biosynthesis of DPA. The *Cg*PS from *C. glutamicum* is commonly regarded as an optimal PS with a high catalytic efficiency for DPA biosynthesis in various hosts including *E. coli* (Tigu et al. 2018; Zhang et al. 2019) and *S. cerevisiae* (Guo et al. 2023), which was considered as a suitable PS candidate for enhancing the DPA production in *Y. lipolytica*. Besides, the abilities of other PSs (*e.g.*, *Yl*PS from *Y. lipolytica*, *Sc*PS from *S. cerevisiae*) to synthesize DPA were also evaluated.

As shown in Fig. 2B, *Cg*PS (strain DPA1-1) produced the highest titer of DPA, reaching 13.7 mg/L in shake-flask fermentation (1.34-fold higher than strain Po1f-HHR), demonstrating the key role of PS in the DPA synthesis in *Y. lipolytica*. Referring to previous reports (Zhang et al. 2019; Guo et al. 2023), these results indicate that *Cg*PS from *C. glutamicum* shows a relatively high catalytic efficiency for DPA biosynthesis in both prokaryotic and eukaryotic hosts, highlighting its suitability as an excellent PS.

### Identify and strengthen the rate-limiting steps in the DPA synthesis pathway

**Fig. 3:**
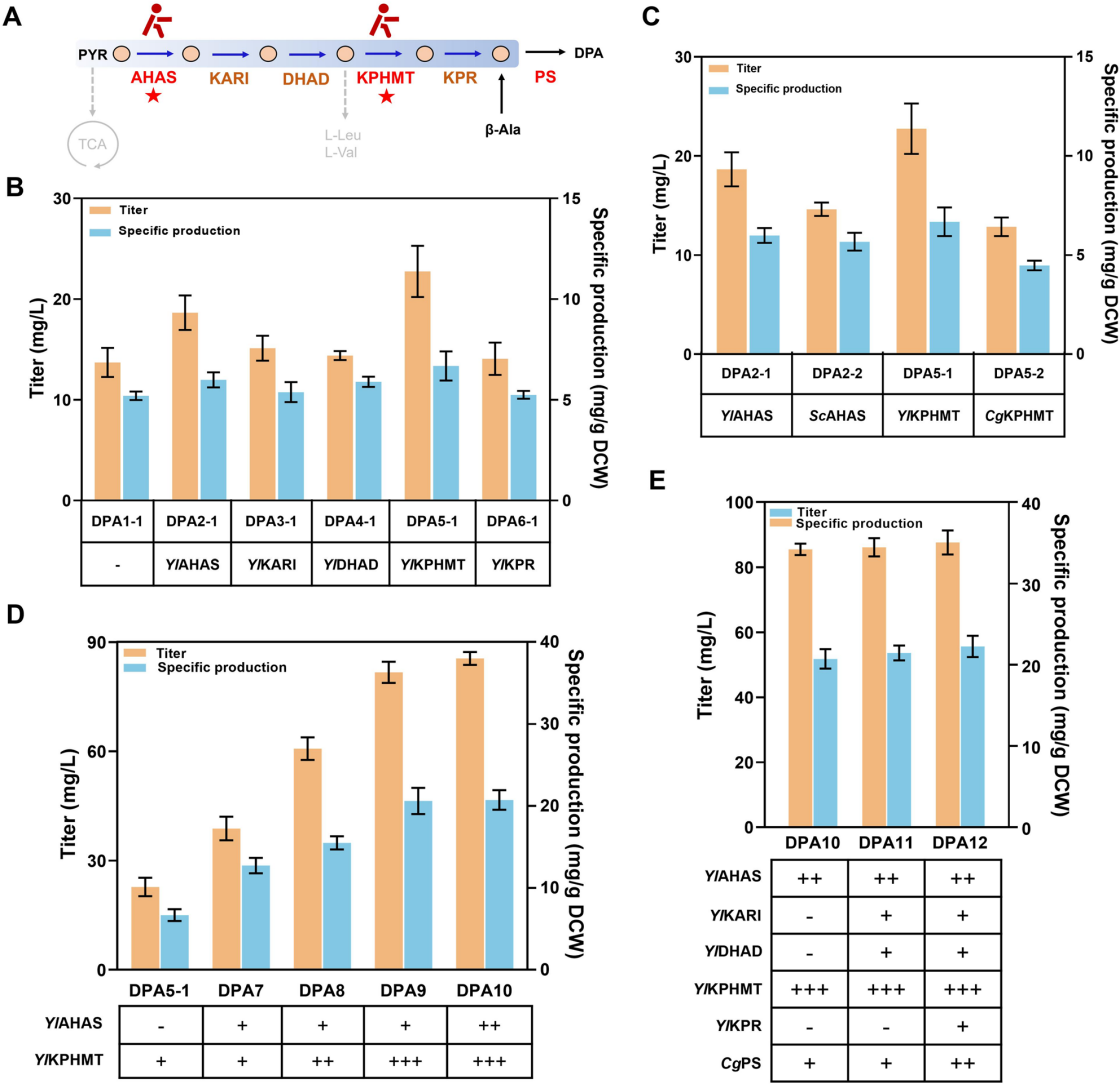
Identification and reinforcement of rate-limiting steps in the DPA pathway of *Y. lipolytica*. (**A**) Pantoic acid biosynthesis pathway (blue background). (**B**) DPA titer and specific production of the strains overexpressing pantoic acid biosynthesis pathway enzymes from *Y. lipolytica*, respectively. (**C**) Comparison of AHASs and KPHMTs from various sources. (**D**) DPA titer and specific production of the strains with different combinations of copy numbers. (**E**) DPA titer and specific production of the strains overexpressing the remaining pathway enzymes. The data represent the means ± standard deviations (*n* = 3)

Pantoic acid (PTA) is the direct precursor of DPA and its biosynthesis starts with pyruvate and proceeds through five sequential enzymatic steps (Fig. 3A) which are catalyzed by acetohydroxyacid synthase (AHAS), ketol-acid reductoisomerase (KARI), dihydroxyacid dehydratase (DHAD), ketopantoate hydroxymethyltransferase (KPHMT), and ketopantoate reductase (KPR), respectively.

Maintaining metabolic flux balance remains a major challenge in multi-gene pathway engineering, primarily due to the accumulation of metabolic intermediates caused by imbalanced expression levels of native pathway genes (Sun et al. 2025). Hence, we overexpressed each enzyme involved in the PTA biosynthesis pathway in strain DPA1-1 to identify the potential rate-limiting steps, relieve the potential bottlenecks, and thereby enhance the metabolic flux towards DPA production.

As shown in Fig. 3B, both strains DPA2-1 (overexpressing *Yl*AHAS) and DPA5-1 (overexpressing *Yl*KPHMT) increased the DPA titers significantly. Among them, strain DPA5-1 reached a higher titer of 22.8 mg/L (1.66-fold higher than strain DPA1-1) and a higher specific production of 6.68 mg/g DCW (1.28-fold higher than strain DPA1-1). Consequently, these two enzymes were identified as the primary rate-limiting steps in the DPA biosynthesis pathway (Fig. 3A).

Heterologous AHAS and KPHMT reported to have high catalytic efficiencies were also investigated (Zhang et al. 2019; Guo et al. 2023), although they did not improve DPA titers significantly in our case (Fig. 3C). This suggests that endogenous AHAS and KPHMT are more suitable for the DPA production in *Y. lipolytica*.

To further strengthen the DPA synthesis pathway, increase of gene copy number is a common strategy to improve the gene expression levels. The optimization of copy numbers of rate-limiting enzymes AHAS and KPHMT was considered to be beneficial for relieving the two enzymatic bottlenecks to enhance the synthesis of DPA.

Therefore, we performed a combinatorial screening of different copy numbers (Fig. 3D). As a result, strain DPA10 (2 copies of *Yl*AHAS and 3 copies of *Yl*KPHMT) finally exhibited the highest titer of 85.5 mg/L (3.75-fold higher than strain DPA5-1) with a specific production of 20.7 mg/g DCW (3.09-fold higher than strain DPA5-1). It was speculated that different expression levels of *Yl*AHAS and *Yl*KPHMT regulate the two rate-limiting steps together. Two copies of *Yl*AHAS pulled more metabolic flux from pyruvate pool, while three copies of *Yl*KPHMT effectively pushed the metabolic flux toward DPA production, which enables them to achieve the maximum DPA synthesis flux.

After optimizing the gene copy number, we co-overexpressed the remaining enzymes (KARI, DHAD, KPR & PS) in the DPA biosynthesis pathway to further reinforce DPA production (Fig. 3E). However, the co-overexpression of them resulted in almost no obvious increase in DPA synthesis, indicating that balanced interactions between the biosynthesis parts are very crucial (Li et al. 2025). This result suggests the presence of additional bottlenecks for DPA biosynthesis.

### Weakening competitive upstream pathways to redirect metabolic flux

**Fig. 4:**
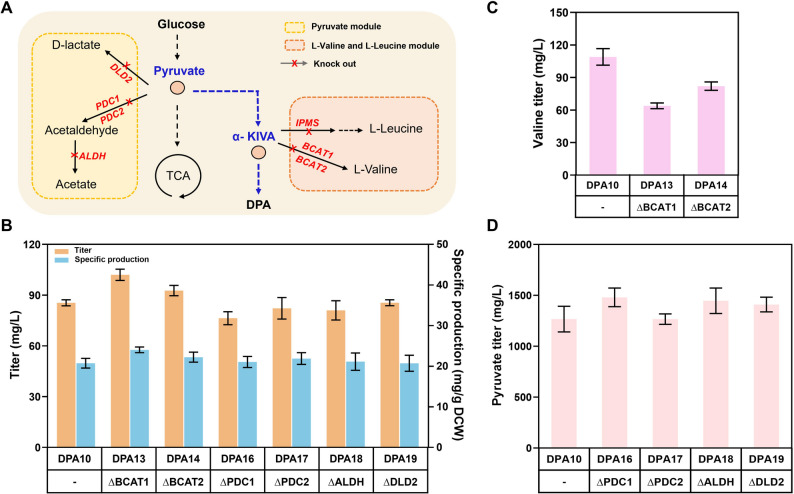
Weakening the competitive upstream pathways redirects metabolic flux in *Y. lipolytica*. (**A**) Competitive upstream pathways. (**B**) DPA titer and specific production of the strains with competitive pathway genes knocked out. (**C**) The L-valine titer of the strains with L-valine biosynthesis genes knocked out. (**D**) The pyruvate titer of the strains with D-Lactic acid and acetate biosynthesis genes knocked out. BACT1/BACT2, branched-chain amino acid aminotransferase; IMPS, 2-isopropylmalate synthase; DLD2, D-2-hydroxyglutarate–pyruvate transhydrogenase; PDC1/PDC2, pyruvate decarboxylase; ALDH, aldehyde dehydrogenase. The data represent the means ± standard deviations (*n* = 3)

Pyruvate and α-KIVA are two key metabolic intermediates for DPA biosynthesis. Pyruvate serves as a central intermediate in complex metabolic network, whereas α-KIVA is involved in L-valine and L-leucine synthesis (Zhang et al. 2021). Thus, metabolic flux diversion may be another bottleneck. Weakening competitive pathways may redirect metabolic flux to produce DPA (Fig. 4A).

After knocking out amino acid aminotransferase (BACT1, BACT2) and 2-isopropylmalate synthase (IPMS) respectively in strain DPA10 (Fig. 4B), which are key enzymes for L-valine and L-leucine synthesis, the DPA titer of strain DPA13 (∆BACT1) reached 102 mg/L (1.19-fold higher than strain DPA10), with a specific production of 27.4 mg/g DCW (1.32-fold higher than strain DPA10). Both the titer and specific production of DPA by strain DPA14 (∆BACT2) increased slightly as compared with DPA10, but were still inferior to those of DPA13 (∆BACT1). Strain DPA15 with IPMS knock-out failed to grow, implying that IPMS (YALI1_B09592g) may be a growth-essential gene in *Y. lipolytica* (Schwartz et al. 2019). As shown in Fig. 4C, a decreased titer of L-valine was observed following the BACT1 and BACT2 knockout, indicating that more of the metabolic flux was redirected to DPA production.

As major metabolites of the pyruvate branched flux, D-lactic acid and acetate can be targeted by weakening their synthesis to effectively enhance pyruvate accumulation (Yin et al. 2024; Lu et al. 2024). Pyruvate decarboxylase (PDC1, PDC2), aldehyde dehydrogenase (ALDH) and D-2-hydroxyglutarate—pyruvate-transhydrogenase (DLD2) were respectively knocked out in strain DPA10 to expand the pyruvate pool. Although a moderate increase was observed in pyruvate accumulation (Fig. 4D), the DPA titer was either not improved significantly or even decreased slightly (Fig. 4B), suggesting that a moderate increase in pyruvate pool may not be sufficient to support further conversion for DPA synthesis in *Y. lipolytica*.

### Inhibiting downstream pathway to promote DPA accumulation

DPA serves as a key precursor for coenzyme A (CoA) synthesis. In *Y. lipolytica*, abundant acetyl-CoA may consume large amounts of DPA and it may be also a negative bottleneck for DPA accumulation (Fig. 1). Reducing its consumption in downstream CoA synthesis pathways may be an efficient approach to enhancing DPA accumulation. Pantothenate Kinase (PanK) catalyzes the phosphorylation of DPA to 4-phosphate pantothenic acid (4PPA), an intermediate that is subsequently converted into CoA (Zhang et al. 2019). Hence, PanK is a key gene for CoA synthesis, which is also a suitable target to be engineered to reduce DPA consumption.

**Fig. 5:**
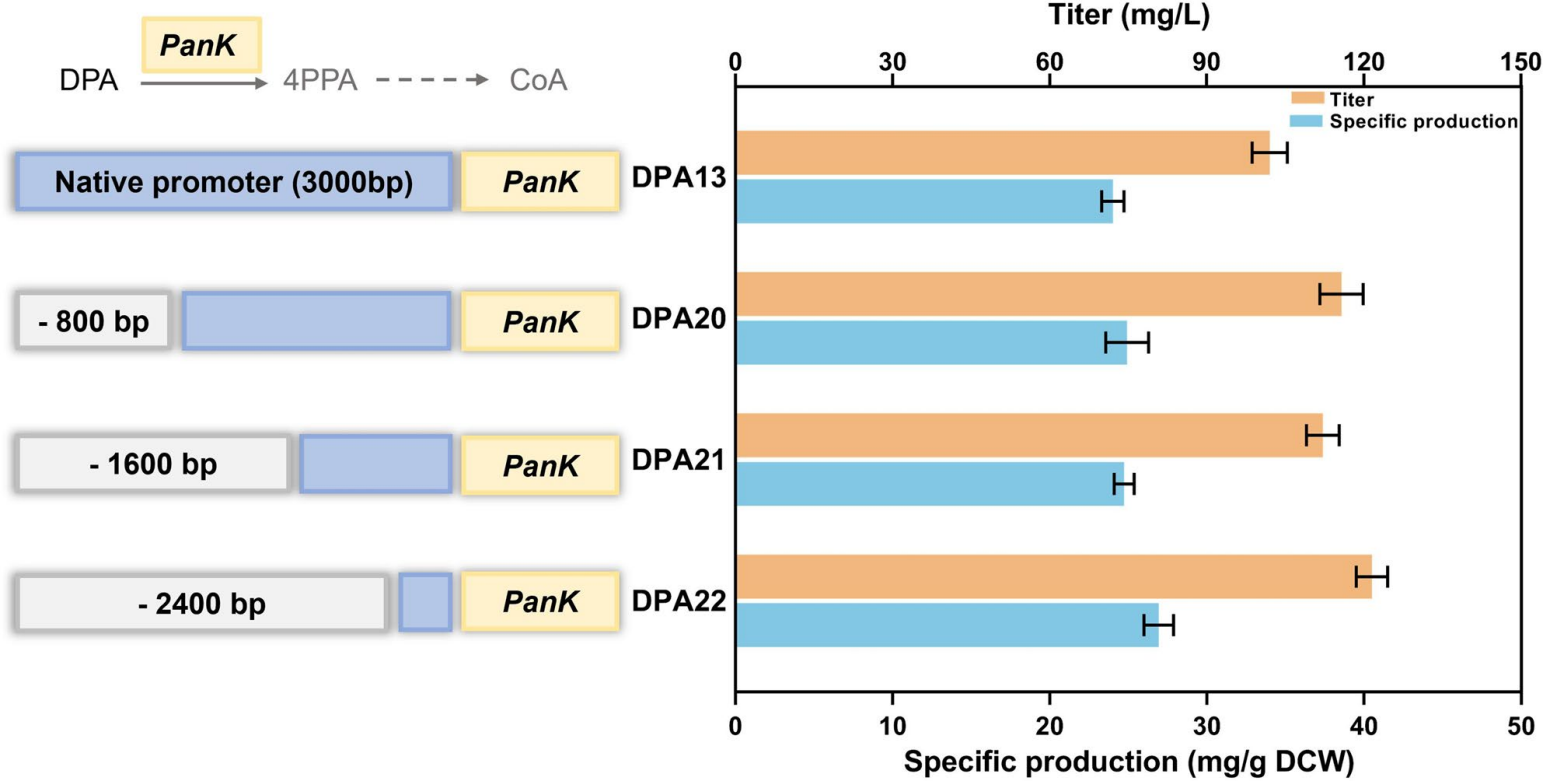
Promoter truncation to enhance DPA accumulation in *Y. lipolytica*. The data represent the means ± standard deviations (*n* = 3)

As CoA is essential for numerous metabolic processes, this pathway should be weakened instead of being knocked out. Promoter truncation is generally used to weaken gene expression level (Cheng et al. 2025b, 2025a). Hence, we truncated the native P_PanK_ promoter in strain DPA13 to downregulate the expression of PanK. As shown in Fig. 5, when comparing the effects of various truncated P_PanK_ promoters on DPA production, a 2,400-bp truncation of P_PanK_ promoter (strain DPA22) led to a 18.6% increase in DPA production as compared with that of strain DPA13, resulting in a higher titer of 121 mg/L.

### Enhancing multiple cofactor supplies to improve biosynthesis efficiency

Metabolic pathway enhancement may lead to a deficiency of cofactors, greatly reducing synthesis capability, and thereby the supply of cofactors may be another bottleneck in DPA biosynthesis. It is necessary to enhance the supply of cofactors (Yue et al. 2024; Zou et al. 2024; Qiu et al. 2025; Zhao et al. 2025b; Chen et al. 2022).

**Fig. 6:**
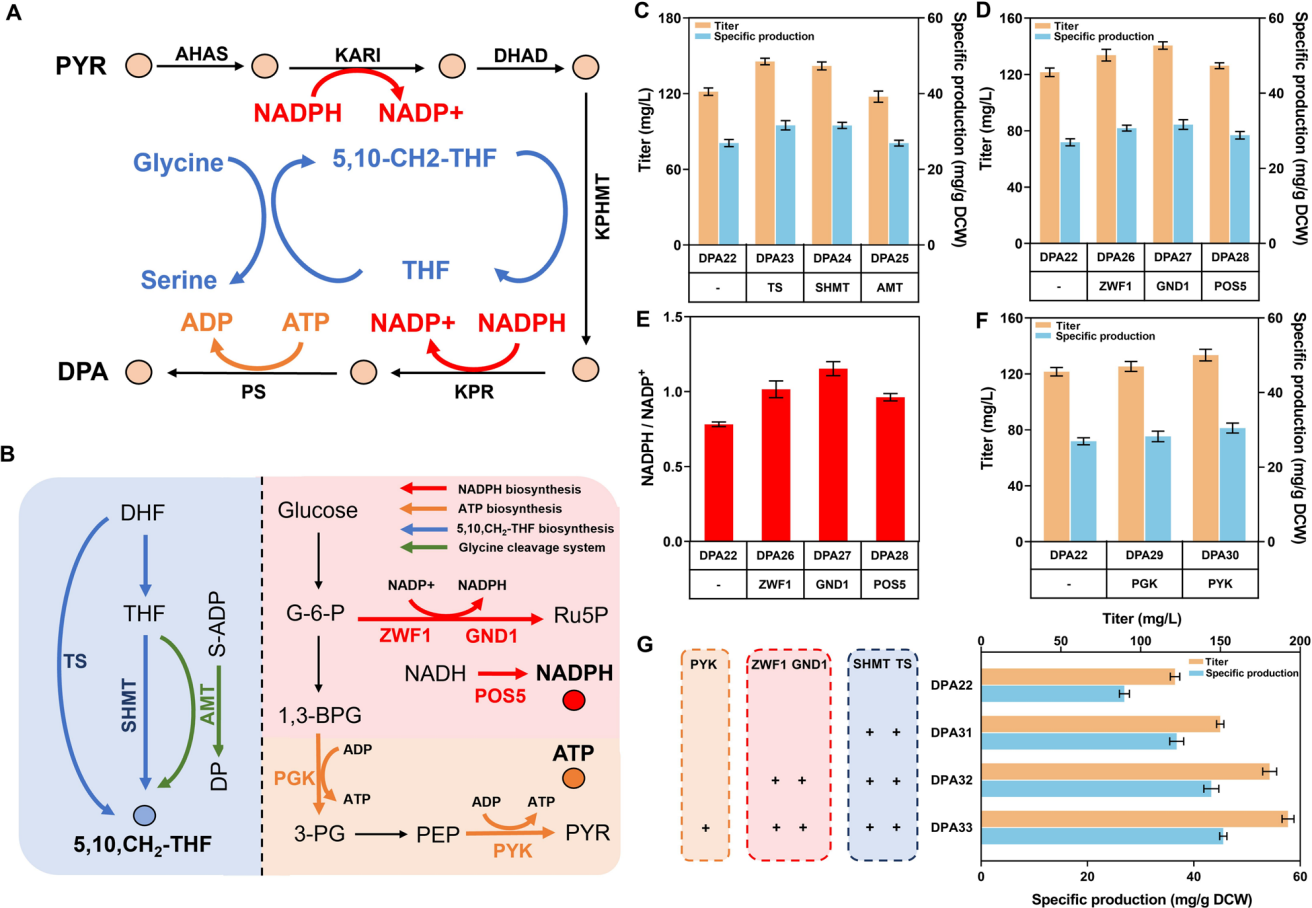
Multi-cofactor supply enhancements for improving biosynthetic efficiency. (**A**) Major cofactors required for the DPA biosynthesis pathway. (**B**) Biosynthesis pathway of cofactors in *Y. lipolytica*. (**C**) DPA titer and specific production of the strains overexpressing relevant enzymes of 5,10-CH_2_-THF biosynthesis. (**D**) DPA titer and specific production of the strains overexpressing relevant enzymes for NADPH biosynthesis. (**E**) Cellular NADPH/NADP.^+^ ratios in the engineered *Y. lipolytica*. strains. (**F**) DPA titer and specific production of the strains overexpressing relevant enzymes for ATP biosynthesis. (**G**) DPA titer and specific production of the strains combined with multi-cofactor supply enhancements. ZWF1, glucose-6-phosphate dehydrogenas; GND1, 6-phosphogluconate dehydrogenase; TS, thymidylate synthase; POS5, NADH kinase; SHMT, hydroxymethyltransferase; AMT, aminomethyltransferase; PGK, phosphoglycerate kinase; PYK, pyruvate kinase; S-ADP, S-aminomethyldihydrolipoylprotein; DP, dihydrolipoylprotein. The data represent the means ± standard deviations (*n* = 3)

Biosynthesis of DPA requires multiple cofactors: 5,10-CH_2_-THF, NADPH and ATP (Fig. 6A). By increasing the expression of key enzymes to enhance the multi-cofactor supply, the entire pathway can be balanced for improved DPA biosynthesis efficiency. Key enzymes involved in the biosynthesis of each cofactor were overexpressed individually in DPA22 (Fig. 6B).

KPHMT catalyzes the transfer of a hydroxymethyl group from 5,10-CH_2_-THF to ketopantoic acid (KPTA). The sufficient supply of 5,10-CH_2_-THF provides adequate hydroxymethyl groups, thereby enhancing the catalytic efficiency of KPHMT. As illustrated in Fig. 6B, 5,10-CH_2_-THF is synthesized from tetrahydrofolate (THF) by serine hydroxymethyltransferase (SHMT), or from glycine by aminomethyltransferase (AMT) (Santatiwongchai et al. 2019; Zou et al. 2024). Additionally, it can also be synthesized from dihydrofolate (DHF) through thymidylate synthase (TS). Hence, SHMT, AMT and TS were overexpressed to enhance the supply of 5,10-CH_2_-THF. As shown in Fig. 6C, the DPA titers of strains DPA23 (overexpressing TS) and DPA24 (overexpressing SHMT) reached 145 mg/L and 141 mg/L, respectively. In contrast, DPA25 (overexpressing AMT) showed a reduced production, which may be caused by a potential metabolic disorder.

NADPH serves as a reduction driving force in biosynthetic reactions and it is primarily generated through the pentose phosphate pathway (PPP). Considering that the biosynthesis pathway requires more NADPH, glucose-6-phosphate dehydrogenase (ZWF1), 6-phosphogluconate dehydrogenase (GND1) and NADH kinase (POS5) were overexpressed to enhance supply of NADPH (Zhang et al. 2025b; Xu et al. 2024). The NADPH engineering resulted in the increases of both DPA titer and NADPH/NADP⁺ ratio (Fig. 6D, E), indicating that enhancing the NADPH supply is an effective strategy for elevating the DPA titer in *Y. lipolytica*. Strain DPA27 (overexpressing GND1) achieved a higher DPA titer of 140 mg/L (1.16-fold higher than strain DPA22), with a specific production of 34.3 mg/g DCW (1.09-fold higher than strain DPA22).

In the DPA biosynthesis pathway, PS requires ATP for the enzymatic condensation reaction to produce DPA. Therefore, overexpressing phosphoglycerate kinase (PGK) and pyruvate kinase (PYK) may balance the ATP consumption. Strain DPA30 (overexpressing PYK) achieved a DPA titer of 132 mg/L, representing 1.09-fold increase compared to DPA22 (Fig. 6F).

Finally, enhancements of multiple cofactor supplies were combined to collaboratively improve the biosynthesis efficiency and increase the DPA titer (Fig. 6G). Consequently, strain DPA33 with enhanced multi-cofactor supply achieved the highest DPA titer of 192 mg/L (or 43.2 mg/g DCW).

### Fed-batch fermentation for high-level DPA production

**Fig. 7:**
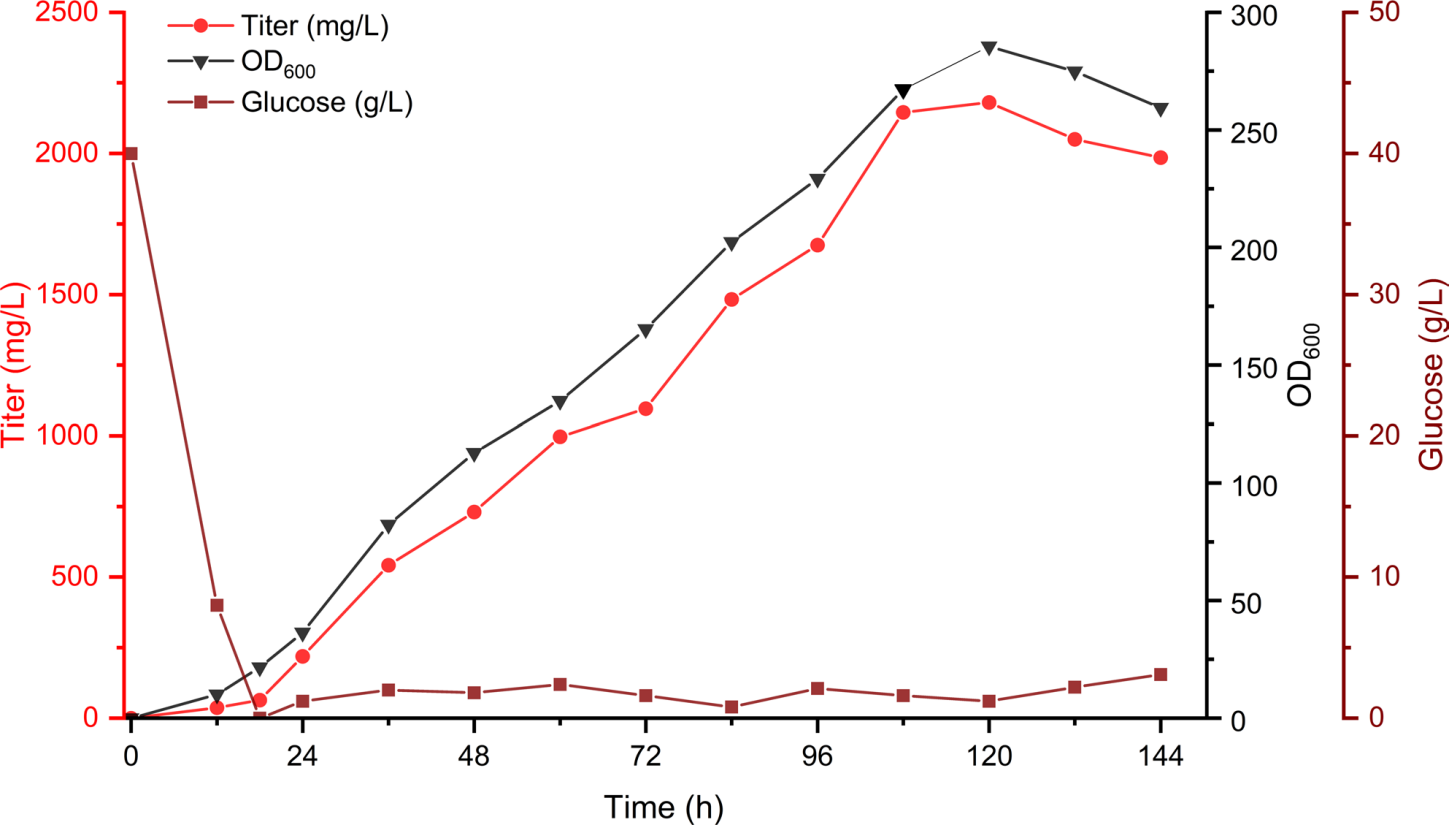
Production of DPA by fed-batch fermentation using the engineered strain DPA34 in a 5-L bioreactor. Samples were withdrawn every 12 h to measure OD600, residual glucose concentrations, and the DPA titer

To evaluate the potential production capacity, the *URA3* and *LEU2* markers of recombinant strain DPA33 were recovered, giving strain DPA34. A two-phase fermentation process was employed for the cell growth (phase I) and DPA production (phase II) (Fig. 7). In phase I, 40 g/L glucose in basal salt medium maintained the cell growth and the biomass reached an OD_600_ of 21.6 when glucose was completely exhausted at 18 h. In phase II, 40 g β-alanine was gradually added to the 5-L bioreactor as the substrate for DPA synthesis. After 24 h, the accumulation of DPA increased significantly, alongside a rapid rise of biomass. After 120 h cultivation, the biomass reached an OD_600_ of 285, and the DPA titer increased up to 2.18 g/L which is the first and highest reported to date for DPA production in *Y. lipolytica*.

## Conclusion

In this study, a *Yarrowia lipolytica* cell factory was designed and constructed to synthesize DPA by implementing various metabolic engineering strategies, such as identifying rate-limiting steps, regulating the expression level of key enzymes, weakening the strongly competitive pathway, and enhancing multiple cofactor supplies. This work is of importance to facilitate the development of *Yarrowia lipolytica* for sustainable production of vitamin B_5_ and its derivatives. However, there is still some work to be done to improve the biosynthesis efficiency of our cell factory and enhance DPA production. For instance, we could further explore transcriptomic analysis and metabolic flux analysis to uncover additional promising genes, utilize protein design strategies to improve the enzymatic activity of key enzymes, balance intracellular β-Ala biosynthesis and DPA biosynthesis to realize the de novo biosynthesis of DPA.

## Supplementary Information

Below is the link to the electronic supplementary material.

Supplementary Material 1

## Data Availability

All data produced and analyzed in the course of this study have been comprehensively incorporated into this article and the accompanying supplementary information file.

## References

[CR1] Acevedo-Rocha CG, Gronenberg LS, Mack M, Commichau FM, Genee HJ (2019) Microbial cell factories for the sustainable manufacturing of B vitamins. Curr Opin Biotechnol 56:18–29. 10.1016/j.copbio.2018.07.006 30138794 10.1016/j.copbio.2018.07.006

[CR2] Barritt SA, DuBois-Coyne SE, Dibble CC (2024) Coenzyme A biosynthesis: mechanisms of regulation, function and disease. Nat Metab 6(6):1008–1023. 10.1038/s42255-024-01059-y 38871981 10.1038/s42255-024-01059-y

[CR3] Cai X, Shi X, Liu S-Q, Qiang Y, Shen J-D, Zhang B et al (2023) Hot spot-based engineering of ketopantoate hydroxymethyltransferase for the improvement of D-pantothenic acid production in *Escherichia coli*. J Biotechnol 364:40–49. 10.1016/j.jbiotec.2023.01.010 36708995 10.1016/j.jbiotec.2023.01.010

[CR4] Chen R, Gao J, Yu W, Chen X, Zhai X, Chen Y et al (2022) Engineering cofactor supply and recycling to drive phenolic acid biosynthesis in yeast. Nat Chem Biol 18(5):520–529. 10.1038/s41589-022-01014-6 35484257 10.1038/s41589-022-01014-6

[CR5] Cheng J, Chen J, Chen D, Li B, Wei C, Liu T et al (2025a) Development of a *Komagataella phaffii* cell factory for sustainable production of ( +)-valencene. Microb Cell Fact 24(1):29. 10.1186/s12934-025-02649-5 39838465 10.1186/s12934-025-02649-5 PMC11752624

[CR6] Cheng J, Chen J, Chen D, Li B, Wen Z, Jin Y et al (2025b) Development of *Komagataella phaffii* as a cell factory for efficient de novo production of β-caryophyllene. New Biotechnol 85:52–58. 10.1016/j.nbt.2024.12.002 10.1016/j.nbt.2024.12.002 39653076

[CR7] Eggersdorfer M, Laudert D, Létinois U, McClymont T, Medlock J, Netscher T et al (2012) One hundred years of vitamins—a success story of the natural sciences. Angew Chem Int Ed 51(52):12960–12990. 10.1002/anie.201205886 10.1002/anie.201205886 23208776

[CR8] Francois JM, Alkim C, Morin N (2020) Engineering microbial pathways for production of bio-based chemicals from lignocellulosic sugars: current status and perspectives. Biotechnol Biofuels 13(1):118. 10.1186/s13068-020-01744-6 32670405 10.1186/s13068-020-01744-6 PMC7341569

[CR9] Fu J, Zaghen S, Lu H, Konzock O, Poorinmohammad N, Kornberg A et al (2024) Reprogramming *Yarrowia lipolytica* metabolism for efficient synthesis of itaconic acid from flask to semipilot scale. Sci Adv 10(32):eadn0414. 10.1126/sciadv.adn0414 39121230 10.1126/sciadv.adn0414 PMC11313960

[CR10] Guo J, Sun X, Yuan Y, Chen Q, Ou Z, Deng Z et al (2023) Metabolic engineering of *Saccharomyces cerevisiae* for vitamin B_5_ production. J Agric Food Chem 71(19):7408–7417. 10.1021/acs.jafc.3c01082 37154424 10.1021/acs.jafc.3c01082

[CR11] He W, Liu M, Yue M, Chen Q, Ye S, Zhou J et al (2025) De novo biosynthesis of chlorogenic acid in *Yarrowia lipolytica* through cis-acting element optimization and NADPH regeneration engineering. J Agric Food Chem 73(10):6081–6091. 10.1021/acs.jafc.4c12056 40025709 10.1021/acs.jafc.4c12056

[CR12] Hüser AT, Chassagnole C, Lindley ND, Merkamm M, Guyonvarch A, Elisáková V et al (2005) Rational design of a *Corynebacterium glutamicum* pantothenate production strain and its characterization by metabolic flux analysis and genome-wide transcriptional profiling. Appl Environ Microbiol 71(6):3255–3268. 10.1128/aem.71.6.3255-3268.2005 15933028 10.1128/AEM.71.6.3255-3268.2005 PMC1151861

[CR13] Li X, Wang Y, Chen X, Eisentraut L, Zhan C, Nielsen J et al (2025) Modular deregulation of central carbon metabolism for efficient xylose utilization in *Saccharomyces cerevisiae*. Nat Commun 16(1):4551. 10.1038/s41467-025-59966-x 40379631 10.1038/s41467-025-59966-x PMC12084563

[CR14] Lu P, Bai R, Gao T, Chen J, Jiang K, Zhu Y et al (2024) Systemic metabolic engineering of *Enterobacter aerogenes* for efficient 2,3-butanediol production. Appl Microbiol Biotechnol 108(1):146. 10.1007/s00253-023-12911-8 38240862 10.1007/s00253-023-12911-8 PMC10798932

[CR15] Mao C, Zheng H, Chen Y, Yuan P, Sun D (2024) Development of a type I-E CRISPR-based programmable repression system for fine-tuning metabolic flux toward D-pantothenic acid in *Bacillus subtilis*. ACS Synth Biol 13(8):2480–2491. 10.1021/acssynbio.4c00256 39083228 10.1021/acssynbio.4c00256

[CR16] Miallot R, Millet V, Galland F, Naquet P (2023) The vitamin B_5_/coenzyme A axis: a target for immunomodulation? Eur J Immunol 53(10):e2350435. 10.1002/eji.202350435 37482959 10.1002/eji.202350435

[CR17] Pastor-Nieto MA, Gatica-Ortega ME, Sánchez-Herreros C, Jiménez-Blázquez E, Martín-Fuentes A, Checa-Recio I et al (2021) Calcium pantothenate is present in cosmetics and may cause allergic contact dermatitis. Contact Dermat 84(3):201–203. 10.1111/cod.13709 10.1111/cod.13709 33015832

[CR18] Qiu K, Song F, Wang K, Zhang H, Yin X, Qin Z et al (2025) Efficient synthesis of vitamin B_5_ in *Escherichia coli* by engineering ketopantoate hydroxymethyltransferase and cofactor supply. J Agric Food Chem 73(10):6030–6039. 10.1021/acs.jafc.4c10027 40014792 10.1021/acs.jafc.4c10027

[CR19] Revuelta JL, Buey RM, Ledesma-Amaro R, Vandamme EJ (2016) Microbial biotechnology for the synthesis of (pro)vitamins, biopigments and antioxidants: challenges and opportunities. Microb Biotechnol 9(5):564–567. 10.1111/1751-7915.12379 27373767 10.1111/1751-7915.12379 PMC4993173

[CR20] Rowicki T, Synoradzki L, Włostowski M (2006) Calcium pantothenate. Part 1. (R,S)-pantolactone technology improvement at the tonnage scale. Ind Eng Chem Res 45(4):1259–1265. 10.1021/ie050774u

[CR21] Santatiwongchai J, Gleeson D, Gleeson MP (2019) Theoretical evaluation of the reaction mechanism of serine hydroxymethyltransferase. J Phys Chem B 123(2):407–418. 10.1021/acs.jpcb.8b10196 30522268 10.1021/acs.jpcb.8b10196

[CR22] Schwartz C, Cheng JF, Evans R, Schwartz CA, Wagner JM, Anglin S et al (2019) Validating genome-wide CRISPR-Cas9 function improves screening in the oleaginous yeast *Yarrowia lipolytica*. Metab Eng 55:102–110. 10.1016/j.ymben.2019.06.007 31216436 10.1016/j.ymben.2019.06.007

[CR23] Song F, Qin Z, Qiu K, Huang Z, Wang L, Zhang H et al (2024) Development of a vitamin B_5_ hyperproducer in *Escherichia coli* by multiple metabolic engineering. Metab Eng 84:158–168. 10.1016/j.ymben.2024.06.006 38942195 10.1016/j.ymben.2024.06.006

[CR24] Su R, Wang T, Bo T, Cai N, Yuan M, Wu C et al (2023) Enhanced production of D-pantothenic acid in *Corynebacterium glutamicum* using an efficient CRISPR–Cpf1 genome editing method. Microb Cell Fact 22(1):3. 10.1186/s12934-023-02017-1 36609377 10.1186/s12934-023-02017-1 PMC9817396

[CR25] Sun M-L, Zou Z, Lin L, Ledesma-Amaro R, Wang K, Ji X-J (2025) Systematic metabolic engineering of *Yarrowia lipolytica* for efficient production of phytohormone abscisic acid. Synth Syst Biotechnol 10(1):165–173. 10.1016/j.synbio.2024.10.004 39552760 10.1016/j.synbio.2024.10.004 PMC11564786

[CR26] Tadi SRR, Nehru G, Allampalli SSP, Sivaprakasam S (2022) Engineering precursor and co-factor supply to enhance D-pantothenic acid production in *Bacillus megaterium*. Bioprocess Biosyst Eng 45(5):843–854. 10.1007/s00449-022-02701-3 35175424 10.1007/s00449-022-02701-3

[CR27] Tigu F, Zhang J, Liu G, Cai Z, Li Y (2018) A highly active pantothenate synthetase from *Corynebacterium glutamicum* enables the production of D-pantothenic acid with high productivity. Appl Microbiol Biotechnol 102(14):6039–6046. 10.1007/s00253-018-9017-2 29737393 10.1007/s00253-018-9017-2

[CR28] Wang Y, Han Y, Liu C, Cao L, Ye Q, Ding C et al (2024) Engineering *Yarrowia lipolytica* to produce L-malic acid from glycerol. ACS Synth Biol 13(11):3635–3645. 10.1021/acssynbio.4c00445 39444231 10.1021/acssynbio.4c00445

[CR29] Xu J, Patassini S, Begley P, Church S, Waldvogel HJ, Faull RLM et al (2020) Cerebral deficiency of vitamin B_5_ (D-pantothenic acid; pantothenate) as a potentially-reversible cause of neurodegeneration and dementia in sporadic Alzheimer’s disease. Biochem Biophys Res Commun 527(3):676–681. 10.1016/j.bbrc.2020.05.015 32416962 10.1016/j.bbrc.2020.05.015

[CR30] Xu M, Xie W, Luo Z, Li C-X, Hua Q, Xu J-H (2023) Improving solubility and copy number of taxadiene synthase to enhance the titer of taxadiene in *Yarrowia lipolytica*. Synth Syst Biotechnol 8(2):331–338. 10.1016/j.synbio.2023.04.002 37215159 10.1016/j.synbio.2023.04.002 PMC10196790

[CR31] Xu M, Yang N, Pan J, Hua Q, Li C-X, Xu J-H (2024) Remodeling the homologous recombination mechanism of *Yarrowia lipolytica* for high-level biosynthesis of squalene. J Agric Food Chem 72(17):9984–9993. 10.1021/acs.jafc.4c01779 38635942 10.1021/acs.jafc.4c01779

[CR32] Yin L, Zhou Y, Ding N, Fang Y (2024) Recent advances in metabolic engineering for the biosynthesis of phosphoenol pyruvate–oxaloacetate–pyruvate-derived amino acids. Molecules 29(12):2893. 10.3390/molecules29122893 38930958 10.3390/molecules29122893 PMC11206799

[CR33] Yuan P, Xu M, Mao C, Zheng H, Sun D (2023) Dynamically regulating glucose uptake to reduce overflow metabolism with a quorum-sensing circuit for the efficient synthesis of D-pantothenic acid in *Bacillus subtilis*. ACS Synth Biol 12(10):2983–2995. 10.1021/acssynbio.3c00315 37664894 10.1021/acssynbio.3c00315

[CR34] Yue M, Liu M, Gao S, Ren X, Zhou S, Rao Y et al (2024) High-level de novo production of (2S)-Eriodictyol in *Yarrowia Lipolytica* by metabolic pathway and NADPH regeneration engineering. J Agric Food Chem 72(8):4292–4300. 10.1021/acs.jafc.3c08861 38364826 10.1021/acs.jafc.3c08861

[CR35] Zehra S, Khan MA (2018) Dietary pantothenic acid requirement of fingerling *Channa punctatus* (Bloch) based on growth, feed conversion, liver pantothenic acid concentration and carcass composition. Aquacult Nutr 24(5):1436–1443. 10.1111/anu.12680

[CR36] Zhang B, Zhang XM, Wang W, Liu ZQ, Zheng YG (2019) Metabolic engineering of *Escherichia coli* for D-Pantothenic acid production. Food Chem 294:267–275. 10.1016/j.foodchem.2019.05.044 31126462 10.1016/j.foodchem.2019.05.044

[CR37] Zhang B, Chen L, Jin J-Y, Zhong N, Cai X, Zou S-P et al (2021) Strengthening the (R)-pantoate pathway to produce D-Pantothenic acid based on systematic metabolic analysis. Food Biosci 43:101283. 10.1016/j.fbio.2021.101283

[CR38] Zhang B, Xiao Y, Zhu Y, Liu C, Zhu L, Zhou J et al (2025a) Enhancing vitamin B_5_ biosynthesis by multimodule optimization and protein engineering. Green Chem. 10.1039/D5GC02458G40756320

[CR39] Zhang N, Huang Z-Y, Li H-P, Li C-X, Xu J-H (2025b) Reprogramming *Komagataella phaffii* for a robust chassis toward efficient de novo biosynthesis of (−)-α-Bisabolol. J Agric Food Chem 73(14):8381–8390. 10.1021/acs.jafc.4c11904 10.1021/acs.jafc.4c11904 40131269

[CR40] Zhao K, Tang H, Zhang B, Zou S, Liu Z, Zheng Y (2023) Microbial production of vitamin B_5_: current status and prospects. Crit Rev Biotechnol 43(8):1172–1192. 10.1080/07388551.2022.2104690 36210178 10.1080/07388551.2022.2104690

[CR41] Zhao K, Gao H, Han M, Zhang B, Liu Z, Zou S et al (2025a) Heterologous integration-assisted metabolic engineering in *Escherichia coli* for elevated D-Pantothenic acid production. Metab Eng 92:161–173. 10.1016/j.ymben.2025.08.003 40803582 10.1016/j.ymben.2025.08.003

[CR42] Zhao X, Fang H, Dong N, Kong K, Zhang J, Fan X et al (2025b) Enhancing vitamin B_12_ production in engineered *Escherichia coli* through cofactor engineering and fermentation media optimization. J Agric Food Chem 73(16):9732–9742. 10.1021/acs.jafc.5c00078 40200544 10.1021/acs.jafc.5c00078

[CR43] Zhong Y, Shang C, Tao H, Hou J, Cui Z, Qi Q (2024) Boosting succinic acid production of *Yarrowia lipolytica* at low pH through enhancing product tolerance and glucose metabolism. Microb Cell Fact 23(1):291. 10.1186/s12934-024-02565-0 39443950 10.1186/s12934-024-02565-0 PMC11515616

[CR44] Zhou H-Y, Tang Y-Q, Peng J-B, Wang S-H, Liu Z-Q, Zheng Y-G (2022) Re-designing *Escherichia coli* for high-yield production of β-alanine by metabolic engineering. Biochem Eng J 189:108714. 10.1016/j.bej.2022.108714

[CR45] Zou S, Zhao K, Tang H, Zhang Z, Zhang B, Liu Z et al (2021) Improved production of D-pantothenic acid in *Escherichia coli* by integrated strain engineering and fermentation strategies. J Biotechnol 339:65–72. 10.1016/j.jbiotec.2021.07.014 34352344 10.1016/j.jbiotec.2021.07.014

[CR47] Zou S, Liu J, Zhao K, Zhu X, Zhang B, Liu Z et al (2024) Metabolic engineering of *Escherichia coli* for enhanced production of D-Pantothenic acid. Bioresour Technol 412:131352. 10.1016/j.biortech.2024.131352 39186986 10.1016/j.biortech.2024.131352

