# Supplementary material for: Metabolic engineering of Yarrowia lipolytica targeting bottlenecks to boost D-Pantothenic acid biosynthesis
Source: Bioresour Bioprocess. 2026 Jan 27;13(1):15. doi: 10.1186/s40643-026-01009-4 (PMC12834876; doi:10.1186/s40643-026-01009-4)
Supplement: Supplementary file 1 — Supplementary Material 1 [file 40643_2026_1009_MOESM1_ESM.docx]

**Supporting Information**

**Metabolic Engineering of *Yarrowia lipolytica* Targeting Bottlenecks to Boost D-Pantothenic Acid Biosynthesis**

**Xing-Kai Li,^1^ Nuo Zhang,^1^ Hai-Peng Li,^1^ Zheng-Yu Huang,^1^ Gao-Yue Niu,^1^ Chen-Yi Sun,^1^ Jian-He Xu^1,2,*^**

1. State Key Laboratory of Bioreactor Engineering, Shanghai Collaborative Innovation Centre for Biomanufacturing, East China University of Science and Technology, Shanghai 200237, China.
2. Shanghai Bioforany Biotechnology Corporation, 471 Guiping Road, Shanghai 200233, China.

*Corresponding author. E-mail: jianhexu@ecust.edu.cn

**Supplementary Tables**

**Table S1. Strains constructed in this study.**

| **Strains** | **Description** | **Source** |
| --- | --- | --- |
| Po1f | ATCC MYA-2613, *ura3-302, leu2-270, xpr2-322, axp-2, leu2-270* | Our Lab. ^[S1]^ |
| Po1f-HHR | Po1f, F-1:: *P_hp4d_-ScRad52-scRad59-T_XPR2_* | Our Lab. ^[S1]^ |
| DPA1-1 | Po1f-HHR, A3:: *P_TEFIN_-CgPS-T_CYC1_* | This study |
| DPA1-2 | Po1f-HHR, A3:: *P_TEFIN_-ScPS-T_CYC1_* | This study |
| DPA1-3 | Po1f-HHR, A3:: *P_TEFIN_-YlPS-T_CYC1_* | This study |
| DPA2-1 | DPA1-1, A08:: *P_TEFIN_-YlAHAS1-T_CYC1_-P_hp4d_- YlAHAS2-T_XPR2_* | This study |
| DPA2-2 | DPA1-1, A08:: *P_TEFIN_-ScAHAS1-T_CYC1_-P_hp4d_- ScAHAS2-T_XPR2_* | This study |
| DPA3-1 | DPA1-1, A08:: *P_TEFIN_-YlKARI-T_CYC1_* | This study |
| DPA4-1 | DPA1-1, A08:: *P_TEFIN_-YlDHAD-T_CYC1_* | This study |
| DPA5-1 | DPA1-1, A08:: *P_TEFIN_-YlKPHMT-T_CYC1_* | This study |
| DPA5-2 | DPA1-1, A08:: *P_TEFIN_-CgKPHMT-T_CYC1_* | This study |
| DPA6-1 | DPA1-1, A08:: *P_TEFIN_-YlKPR-T_CYC1_* | This study |
| DPA7 | DPA5-1, POX3:: *P_TEFIN_-YlAHAS1-T_CYC1_-P_hp4d_- YlAHAS2-T_XPR2_* | This study |
| DPA8 | DPA7, AXP:: *P_TEFIN_-YlKPHMT-T_CYC1_* | This study |
| DPA9 | DPA8, E1-3:: *P_TEFIN_-YlKPHMT-T_CYC1_* | This study |
| DPA10 | DPA9, F1-3:: *P_TEFIN_-YlAHAS1-T_CYC1_-P_hp4d_- YlAHAS2-T_XPR2_* | This study |
| DPA11 | DPA10, A1-2:: *P_TEFIN_-YlKARI-(GS)_4_-YlDHAD-T_CYC1_* | This study |
| DPA12 | DPA11, POX6:: *P_TEFIN_-CgPS-P2A-YlKPR-T_CYC1_* | This study |
| DPA13 | DPA10, ΔBACT1 | This study |
| DPA14 | DPA10, ΔBACT2 | This study |
| DPA15 | DPA10, ΔIMPS | This study |
| DPA16 | DPA10, ΔPDC1 | This study |
| DPA17 | DPA10, ΔPDC2 | This study |
| DPA18 | DPA10, ΔALDH | This study |
| DPA19 | DPA10, ΔDLD2 | This study |
| DPA20 | DPA13, ΔtP_pank_(800bp) | This study |
| DPA21 | DPA13, ΔtP_pank_(1600bp) | This study |
| DPA22 | DPA13, ΔtP_pank_(2400bp) | This study |
| DPA23 | DPA22, POX4:: *P_TEFIN_-YlTS-T_CYC1_* | This study |
| DPA24 | DPA22, POX4:: *P_TEFIN_-YlSHMT-T_CYC1_* | This study |
| DPA25 | DPA22, POX4:: *P_TEFIN_-YlAMT-T_CYC1_* | This study |
| DPA26 | DPA22, POX4:: *P_TEFIN_-YlZWF1-T_CYC1_* | This study |
| DPA27 | DPA22, POX4:: *P_TEFIN_-YlGND1-T_CYC1_* | This study |
| DPA28 | DPA22, POX4:: *P_TEFIN_-YlPOS5-T_CYC1_* | This study |
| DPA29 | DPA22, POX4:: *P_TEFIN_-YlPGK-T_CYC1_* | This study |
| DPA30 | DPA22, POX4:: *P_TEFIN_-YlPYK-T_CYC1_* | This study |
| DPA31 | DPA22, POX4:: *P_TEFIN_-YlSHMT-T_CYC1_*-*P_hp4d_-YlTS-T_XPR2_* | This study |
| DPA32 | DPA31, D17:: *P_TEFIN_-YlZWF1-T_CYC1_*-*P_hp4d_-YlGND1-T_XPR2_* | This study |
| DPA33 | DPA32, MFE1:: *P_TEFIN_-YlPYK-T_CYC1_* | This study |
| DPA34 | DPA33, *P_LEU_-LEU-T_LEU_-P_URA3_-URA3-T_URA3_* | This study |

**Table S2. List of main genes used in this study**

| **Genes** | **Source organism** | **Accession numbers** |
| --- | --- | --- |
| *Cg*PS^a^ | *C. glutamicum* | Q9X713 |
| *Yl*PS | *Y. lipolytica* | QNP97301 |
| *Sc*PS^a^ | *S. cerevisiae* | P40459 |
| *Yl*AHAS1 | *Y. lipolytica* | QNP97555 |
| *Yl*AHAS2 | *Y. lipolytica* | QNP96530 |
| *Sc*AHAS1^a^ | *S. cerevisiae* | AJQ44975 |
| *Sc*AHAS1^a^ | *S. cerevisiae* | AJS66334 |
| *Yl*KPHMT | *Y. lipolytica* | QNP97049 |
| *Cg*KPHMT ^a^ | *C. glutamicum* | BAB97507 |
| *Yl*KARI | *Y. lipolytica* | QNP98626 |
| *YlDHAD* | *Y. lipolytica* | QNP96049 |
| *Yl*KPR | *Y. lipolytica* | QNP99333 |

^a^Codon optimized for *Y. lipolytica*

AHAS, acetohydroxyacid synthase; KARI, ketol-acid reductoisomerase;

DHAD, dihydroxyacid dehydratase; KPHMT, ketopantoate hydroxymethyltransferase;

KPR, ketopantoate reductase; PS, pantothenate synthetase;

**Table S3. Plasmids constructed in this study**

| **Plasmids** | **Description** | **Source** |
| --- | --- | --- |
| Sg-A3 | SgRNA plasmids for genome integration. | Our lab. ^[S1]^ |
| Sg-A08 |  |  |
| Sg-AXP |  |  |
| Sg-A1-2 |  |  |
| Sg-POX3 |  |  |
| Sg-POX4 |  |  |
| Sg-POX6 |  |  |
| Sg-F1-3 |  |  |
| Sg-E1-3 |  |  |
| Sg-D17 |  |  |
| Sg-MFE1 |  |  |
| pHR-A3 | Donor plasmids with  upstream (∼1000 bp)  and downstream  (∼1000 bp) homology  arm sequences | Our lab. ^[S1]^ |
| pHR-A08 |  |  |
| pHR-AXP |  |  |
| pHR-A1-2 |  |  |
| pHR-POX3 |  |  |
| pHR-POX4 |  |  |
| pHR-POX6 |  |  |
| pHR-F1-3 |  |  |
| pHR-E1-3 |  |  |
| pHR-D17 |  |  |
| pHR-MFE1 |  |  |
| pHR-A3-1 | *P_TEFIN_-CgPS-T_CYC1_* | This study |
| pHR-A3-2 | *P_TEFIN_-ScPS-T_CYC1_* | This study |
| pHR-A3-3 | *P_TEFIN_-YlPS-T_CYC1_* | This study |
| pHR-A08-1 | *P_TEFIN_-YlAHAS1-T_CYC1_-P_hp4d_- YlAHAS2-T_XPR2_* | This study |
| pHR-A08-2 | *P_TEFIN_-ScAHAS1-T_CYC1_-P_hp4d_- ScAHAS2-T_XPR2_* | This study |
| pHR-A08-3 | *P_TEFIN_-YlKARI-T_CYC1_* | This study |
| pHR-A08-4 | *P_TEFIN_-YlDHAD-T_CYC1_* | This study |
| pHR-A08-5 | *P_TEFIN_-YlKPHMT-T_CYC1_* | This study |
| pHR-A08-6 | *P_TEFIN_-CgKPHMT-T_CYC1_* | This study |
| pHR-A08-7 | *P_TEFIN_-YlKPR-T_CYC1_* | This study |
| pHR-POX3-1 | *P_TEFIN_-YlAHAS1-T_CYC1_-P_hp4d_- YlAHAS2-T_XPR2_* | This study |
| pHR-AXP-1 | *P_TEFIN_-YlKPHMT-T_CYC1_* | This study |
| pHR-E1-3-1 | *P_TEFIN_-YlKPHMT-T_CYC1_* | This study |
| pHR-F1-3-1 | *P_TEFIN_-YlAHAS1-T_CYC1_-P_hp4d_- YlAHAS2-T_XPR2_* | This study |
| pHR-A1-2 | *P_TEFIN_-YlKARI-(GS)_4_-YlDHAD-T_CYC1_* | This study |
| pHR-POX6-1 | *P_TEFIN_-CgPS-P2A-YlKPR-T_CYC1_* | This study |
| Sg-ΔBACT1 | SgRNA plasmids for gene knockout | This study |
| Sg-ΔBACT2 |  |  |
| Sg-ΔIMPS |  |  |
| Sg-ΔPDC1 |  |  |
| Sg-ΔPDC2 |  |  |
| Sg-ΔALDH |  |  |
| Sg-ΔDLD2 |  |  |
| Sg-ΔtP_pank_ |  |  |
| pHR-ΔBACT1 | Donor plasmids with  upstream (∼1000 bp)  and downstream  (∼1000 bp) homology  arm sequences | This study |
| pHR-ΔBACT2 |  |  |
| pHR-ΔIMPS |  |  |
| pHR-ΔPDC1 |  |  |
| pHR-ΔPDC2 |  |  |
| pHR-ΔALDH |  |  |
| pHR-ΔDLD2 |  |  |
| pHR-ΔtP_pank-800_ |  |  |
| pHR-ΔtP_pank-1600_ |  |  |
| pHR-ΔtP_pank-2400_ |  |  |
| pHR-POX4-1 | *P_TEFIN_-YlTS-T_CYC1_* | This study |
| pHR-POX4-2 | *P_TEFIN_-YlSHMT-T_CYC1_* | This study |
| pHR-POX4-3 | *P_TEFIN_-YlAMT-T_CYC1_* | This study |
| pHR-POX4-4 | *P_TEFIN_-YlZWF1-T_CYC1_* | This study |
| pHR-POX4-5 | *P_TEFIN_-YlGND1-T_CYC1_* | This study |
| pHR-POX4-6 | *P_TEFIN_-YlPOS5-T_CYC1_* | This study |
| pHR-POX4-7 | *P_TEFIN_-YlPGK-T_CYC1_* | This study |
| pHR-POX4-8 | *P_TEFIN_-YlPYK-T_CYC1_* | This study |
| pHR-POX4-9 | *P_TEFIN_-YlTS-T_CYC1_*-*P_hp4d_-YlSHMT-T_XPR2_* | This study |
| pHR-D17-1 | *P_TEFIN_-YlZWF1-T_CYC1_*-*P_hp4d_-YlGND1-T_XPR2_* | This study |
| pHR-MFE-1 | *P_TEFIN_-YlPYK-T_CYC1_* | This study |
| HB | *P_LEU_-LEU-T_LEU_-P_URA3_-URA3-T_URA3_* | This study |

**Table S4. List of primers used in this study.**

| **Primer** | **Sequence (5’-3’)** |
| --- | --- |
| *CgPS*-F | accagcactttttgcagtactaaccgcagatgcaggtggcaaccaccaagcagg |
| *CgPS*-R | gtaagcgtgacataactaattacatgatcagagctcaatgttgtcaatcagc |
| *ScPS*-F | accagcactttttgcagtactaaccgcagatgaagatcttccacaccgtggagg |
| *ScPS*-R | gtaagcgtgacataactaattacatgatcaaatgactatgttgtcgatcag |
| *YlPS*-F | accagcactttttgcagtactaaccgcagatgttgcgaccggtgattcggctag |
| *YlPS*-R | gtaagcgtgacataactaattacatgattaacacagaacattgtcaatgattc |
| *YlAHAS*-F-1 | accagcactttttgcagtactaaccgcagatgcaatcgcgactcgcccg |
| *YlAHAS*-R-1 | gcatggatccagagccgctgcctgaaccgttctcaaaggtggtct |
| *YlAHAS*-F-2 | ggttcaggcagcggctctggatccatgcttggaaaacgatttgtgggt |
| *YlAHAS*-R-2 | tgtaagcgtgacataactaattacatgactaaccaggaggcagagaggtctgg |
| *ScAHAS*-F-1 | gcactttttgcagtactaaccgcagatgatccgacagtcgactttga |
| *ScAHAS*-R-1 | gcagcatggatccagagccgctgcctgaaccatgcttaccgccggttcg |
| *ScAHAS*-F-2 | ggttcaggcagcggctctggatccatgctgcgatcgctactgcaatcg |
| *ScAHAS*-R-2 | tgtaagcgtgacataactaattacatgactaacccgggggaagctggg |
| *YlKARI*-F | ccagcactttttgcagtactaaccgcagatgtctgcccgacttttctccactg |
| *YlKARI*-R | agggcgtgaatgtaagcgtgacataactaattacatgattagttgttctcgggtcgcagctttc |
| *YlDHAD*-F | accagcactttttgcagtactaaccgcagatgattcgagcacgaaatcaggctatg |
| *YlDHAD*-R | cgtgaatgtaagcgtgacataactaattacatgattaggcgtcagtgatacagccagt |
| *YlKPHMT*-F | accagcactttttgcagtactaaccgcagatgtctcttttgcgcactctgaggcccg |
| *YlKPHMT*-R | cgtgaatgtaagcgtgacataactaattacatgattatttcagtttagccgcttcctcatcaccca |
| *YlKPR*-F | ctttttgcagtactaaccgcagatgagtttaccgaaagttcacattctgggagcaggttca |
| *YlKPR*-R | agggcgtgaatgtaagcgtgacataactaattacatgattaaaacttgttgaagtcctgctgtgc |
| *CgKPHMT*-F | accagcactttttgcagtactaaccgcagatgcctatgtcgggcattgacgc |
| *CgKPHMT*-R | agggcgtgaatgtaagcgtgacataactaattacatgatcagaatgactcagcctctcctggga |
| *ZWF1-*F | cagcactttttgcagtactaaccgcagatgactggcaccttacccaagttcg |
| *ZWF1*-R | gaatgtaagcgtgacataactaattacatgatcacgaggagcccttggtgaca |
| *POS5-*F | cagcactttttgcagtactaaccgcagatgcgactactcatccgccgaacc |
| *POD5-*R | gaatgtaagcgtgacataactaattacatgactaagcaacatcgcctgacgcct |
| *GND1-*F | cagcactttttgcagtactaaccgcagatgactgacacttcaaacatcaagtgag |
| *GND1-*R | gcgtgaatgtaagcgtgacataactaattacatgattaagcatcgtaagtggaagaagaaacct |
| *PYK-*F | agcactttttgcagtactaaccgcagatgatttacaccgccaattcgtccccttc |
| *PYK*-R | gtgaatgtaagcgtgacataactaattacatgattagacacactcgagaactcggagagt |
| *PGK-*F | agcactttttgcagtactaaccgcagatgtctcttaccaacaagctctccatc |
| *PGK-*R | gtgaatgtaagcgtgacataactaattacatgattacttcttctcggagagagcagcga |
| *TS-*F | accagcactttttgcagtactaaccgcagatgaccactctcaacgaacaaggatacct |
| *TS-*R | gtgaatgtaagcgtgacataactaattacatgactaaacgctcatctgcatctcaatcttgg |
| *SHMT-*F | accagcactttttgcagtactaaccgcagatgcagcgattcactcgactttcccg |
| *SHMT-*R | tgaatgtaagcgtgacataactaattacatgactatttgtatccagcaggatcaatagggaaagggt |
| *AMT-*F | accagcactttttgcagtactaaccgcagatgcttcgagttgcacacttttccc |
| *AMT-*R | tgaatgtaagcgtgacataactaattacatgactacttgaagtacttgtggggaacaaagg |
| X-pHR-F | tcatgtaattagttatgtcacgcttacattcacgc |
| X-pHR-R | ctgcggttagtactgcaaaaagtgctggt |
| sg-ΔtPpank-F | acgtgtgctaaagagacaccaggggttttagagctagaaatagcaagttaaaataaggc |
| sg-ΔtPpank-R | ctaaaacccctggtgtctctttagcacacgtcaacctgcgccga |
| sg-ΔBCAT1-F | acgttacctcggtgagaagcacaagttttagagctagaaatagcaagttaaaataaggc |
| sg-ΔBCAT1-R | ctaaaacttgtgcttctcaccgaggtaacgtcaacctgcgccga |
| sg-ΔBCAT2-F | acgtcgacgagggcaacatcaccggttttagagctagaaatagcaagttaaaataaggc |
| sg-ΔBCAT2-R | ctaaaaccggtgatgttgccctcgtcgacgtcaacctgcgccga |
| sg-ΔIPMS-F | acgtggtgaccaacaagtccaagggttttagagctagaaatagcaagttaaaataaggc |
| sg-ΔIMPS-R | ctaaaacccttggacttgttggtcaccacgtcaacctgcgccga |
| sg-ΔPDC1-F | acgtcggtacggaggaacatacgtgttttagagctagaaatagcaagttaaaataaggc |
| sg-ΔPDC1-R | cacgtatgttcctccgtaccgacgtcaacctgcgccgacccg |
| sg-ΔPDC2-F | acgtgctgccatgtcatatccacggttttagagctagaaatagcaagttaaaataaggc |
| sg-ΔPDC2-R | tagctctaaaaccgtggatatgacatggcagcacgtcaacctgcgccg |
| sg-ΔALDH-F | acgttgtcaaggccatgagcaccggttttagagctagaaatagcaagttaaaataaggc |
| sg-ΔALDH-R | ctaaaaccggtgctcatggccttgacaacgtcaacctgcgccga |
| sg-ΔDLD2-F | acgtaggtcaagcagactgccgaggttttagagctagaaatagcaagttaaaataaggc |
| sg-ΔDLD2-R | ctaaaacctcggcagtctgcttgacctacgtcaacctgcgccga |
| ΔtP_pank-800_-F-Z | aggtaccaaggaagcatgcggtaccaactcttaatctgtcgctgcctggac |
| ΔtP_pank-800_-R-Z | atgcaagacctgctagtgacatttccaaattgtttggcgacttatga |
| ΔtP_pank-1600_-F-Z | aggtaccaaggaagcatgcggtacgatcctcagatgcatacagaccagagca |
| ΔtP_pank-1600_-R-Z | gcgatgcaagacctgctaggccccgttatctcatttaaccccaga |
| ΔtP_pank-2400_-F-Z | aggtaccaaggaagcatgcggtacgattgtcttgaagacgagacatgaggccct |
| ΔtP_pank-2400_-R-Z | cgatgcaagacctgctaggagctagagctagttggcatggtatgga |
| ΔtP_pank_-F-Y | ctagcaggtcttgcatcgctccttc |
| ΔtP_pank_-R-Y | cagctatgaccatgattacgccaagcttacaacagttgcagtaagaagtcaaaaccg |
| HR-ΔBCAT1-F | aggtaccaaggaagcatgcggtacaacggggcaagtagggttaatagc |
| HR-ΔBCAT1-R | cagctatgaccatgattacgccaagcttccaacgcaagctctgcaaaaaga |
| HR-ΔBCAT2-F | aggtaccaaggaagcatgcggtaccgtgcacggtttttattggaga |
| HR-ΔBCAT2-R | cagctatgaccatgattacgccaagcttcacaggactcactcagctgatcc |
| HR-ΔIMPS-F | aggtaccaaggaagcatgcggtacctcatgtaccgggacaatagagtttg |
| HR-ΔIMPS-R | cagctatgaccatgattacgccaagcttaccttttctgtagcatttctcaaccgac |
| HR-ΔPDC1-F | aggtaccaaggaagcatgcggtaccatagatgagagagtaaacagcgcaa |
| HR-ΔPDC1-R | tatgaccatgattacgccaagcttctccatatcaacctacaagtacaagtacccca |
| HR-ΔPDC2-F | aggtaccaaggaagcatgcggtacagctctgaaggctacacaatctaacc |
| HR-ΔPDC2-R | cagctatgaccatgattacgccaagcttgtatgtacgatagtcgtctgatcaggt |
| HR-ΔALDH-F | aggtaccaaggaagcatgcggtacatcatctgatcatctcttgtttggttcttctttct |
| HR-ΔALDH-R | cagctatgaccatgattacgccaagcttttgcggcctttttaatcacatttgtcatgt |
| HR-ΔDLD2-F | aggtaccaaggaagcatgcggtaccgccgccacctggggt |
| HR-ΔDLD2-R | cagctatgaccatgattacgccaagcttgcgtaacgagtctgcgagct |

**Supplementary Figures**

**Figure S1.**

**
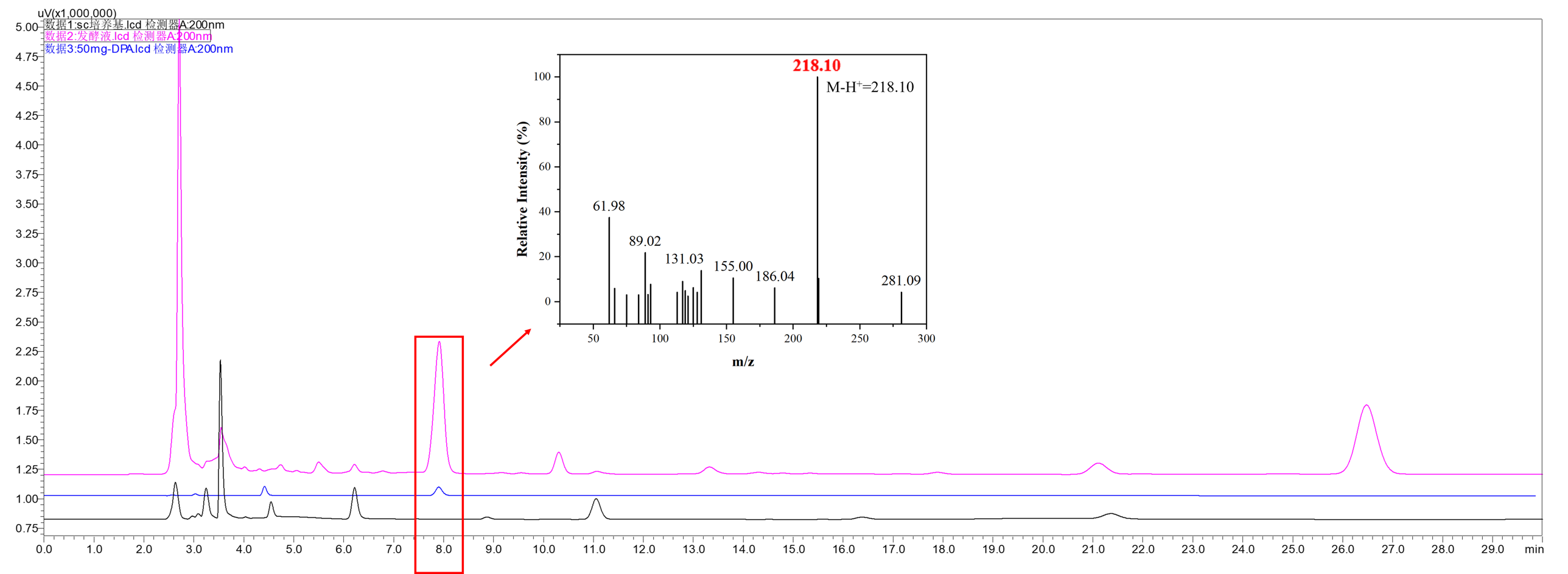
**

**Figure S1.** DPA levels in the engineered strain were determined by HPLC and LC-MS.

(Blue line, DPA standard; pink line, fermentation broth supernatant; black line, SC medium)

**Supplementary reference**

（S1）Xu, M., Yang, N., Pan, J., Hua, Q., Li, C.-X., & Xu, J.-H. (2024). Remodeling the Homologous Recombination Mechanism of *Yarrowia lipolytica* for High-Level Biosynthesis of Squalene. J. Agric. Food Chem., 72(17), 9984-9993. https://doi.org/10.1021/acs.jafc.4c01779
